# Protocol for the impact of paramedics in NHS primary care: application of realist approaches to improve understanding and support intelligent policy and future workforce planning

**DOI:** 10.29045/14784726.2019.12.4.3.35

**Published:** 2019-12-01

**Authors:** Georgette Eaton, Veronika Williams, Geoff Wong, Nia Roberts, Kamal R. Mahtani

**Affiliations:** University of Oxford; London Ambulance Service NHS Trust: ORCID iD: http://orcid.org/0000-0001-9421-2845; Nipissing University; University of Oxford: ORCID iD: https://orcid.org/0000-0001-5660-8224; University of Oxford: ORCID iD: https://orcid.org/0000-0002-5384-4157; University of Oxford; University of Oxford: ORCID iD: https://orcid.org/0000-0002-7791-8552

**Keywords:** paramedics, primary care, realist approaches, urgent care

## Abstract

**Introduction:**

In the United Kingdom, changing demands on ambulance services has caused a change in what is expected of a paramedic. As well as advanced life support, paramedics now need to be skilled in managing a range of urgent case presentations, with emphasis on treat-at-scene. The change in the scope of work paramedics can undertake has established their role within primary care. However, as paramedics transition to roles within primary care, their knowledge and skillset will undoubtedly need to change. The current opportunities for paramedics’ employment in primary care require careful evaluation. In order to contribute to patients’ and the NHS’ primary care agenda, evidence must be generated to show how and why these changes would work, for whom, in what context and to what extent.

**Methods and analysis:**

The purpose of this research is to produce findings that will improve our understanding of the ways in which (i.e. how, why and in what contexts) paramedics impact on the primary care workforce. A theory-driven approach to evidence-synthesis will be conducted in a realist review, to produce a programme theory. This programme theory will be tested using empirical data collected through a realist evaluation. Survey and interview data will be collected from paramedics working in primary care, general practitioners and patients to assess under which contexts, and by which mechanisms, paramedics are working in primary care, and thus test the programme theory. Based on the findings, we will be able to highlight the role of paramedics in primary care, as well as how they operate and under what conditions.

**Ethics and dissemination:**

Formal ethics review is not required for the review, as it is secondary research, but will be sought for the evaluation. Findings will be disseminated in a peer-reviewed journal, at national and international conferences and to relevant professional associations.

## Introduction

In the United Kingdom, paramedics have traditionally provided emergency care within an ambulance service, responding to life-threatening emergencies through the 999-call system. However, the role of the ambulance service has changed substantially, and the role of the paramedic has subsequently evolved. Now, just 8% of 999 calls are for people with life-threatening illnesses or injuries (Keogh, 2017), indicating that a large proportion of patients access the ambulance service with lower acuity presentations.

This has subsequently caused a change in what is expected of a paramedic. As well as advanced life support, paramedics now need to be skilled in managing acute-on-chronic long-term conditions, acute presentations of mental ill-health, social-care assessments and a range of urgent care presentations (National Institute for Health and Care Excellence, 2017a, 2017b, 2018). New legislation now also supports non-medical prescribing by advanced paramedics, allowing these paramedics to ‘complete’ patient care and avoid referrals for secondary assessments and treatment. With changing demands on the health and social service, higher education thresholds and social dependence on the ambulance service (Wankhade, 2010), the 21st-century paramedic has evolved to be a generalist.

Opportunities for paramedics to work in other healthcare settings have prompted a migration of paramedics from their traditional employer, the NHS ambulance services, to settings including acute hospital trusts, forensic healthcare, minor injury units, general practice services and urgent care centres (Evans, McGovern, Birch, & Newbury-Birch, 2014; Mahtani, Eaton, Catterall, & Ridley, 2018; O’Meara, 2014; Williams, Fielder, Strong, Acker, & Thompson, 2015). The NHS Long Term Plan (NHS England, 2019) further advocates the use of paramedics in primary care. With an increased demand in services, and more patients requiring complex case management within the community, it is unsurprising that primary care services are facing unprecedented challenges (Montgomery et al., 2017). These challenges are leading to recruitment and retention issues for doctors within primary care (Majeed, 2017), opening up opportunities for paramedics to work in this setting. Attracted to ‘normal hours’ and an opportunity to further develop their professional practice (Eaton, Mahtani, & Catterall, 2018), this new ability of paramedics to manage low acuity and complex case presentations appears to be well suited to primary care.

As paramedics transition to roles within primary care, their knowledge and skillset will need to change (National Institute for Health and Care Excellence, 2017a, 2017b; Primary Care Workforce Commission, 2015). Therefore, the current opportunities for paramedics’ employment in primary care requires careful evaluation in order to contribute to patients’ and the NHS’ primary care agenda. Evidence must be generated to show how and why these changes would work, for whom, in what context and to what extent. Gaining a comprehensive understanding of why paramedics are opting for these roles, how they practise in this setting and what their contribution to patient care is will contribute to the development of the profession, workforce planning and patient safety. Without this understanding, development of a future workforce that is fit for purpose will lack direction.

## Methods and analysis

This research aims to improve our understanding of the ways in which (i.e. how, why and in what contexts) paramedics impact (or not) on the primary care workforce.

### Research objectives

1. To conduct a realist review to understand the ways in which paramedics impact on the primary care workforce. This will be done with: (a) engagement with a diverse range of literature; (b) the development of a programme theory; and (c) feedback and advice from stakeholders experienced in the field.
2. To undertake a realist evaluation of the existing ways in which paramedics work in primary care within the NHS. This will build on the information obtained from the realist review and develop the programme theory.
3. To produce recommendations that guide the implementation of a workplace-based framework and curriculum for paramedics working in primary care.

### Research questions

What is the role of paramedics working in NHS primary care?

#### Sub questions

1. How, why, for whom and in what contexts do paramedics ‘work’ in primary care settings?
2. What is the impact of paramedics working in primary care teams on the working practices of other healthcare professionals and the experiences of patients?
3. What knowledge, capabilities and skills do paramedics working in primary care need to work in primary care within the NHS?

### Study design

This research has two main approaches: realist review (Work Package 1) and realist evaluation (Work Package 2). Our research design is based on the work of Pawson, Greenhalgh, Harvey and Walshe (2005), and follows the standards for conducting and reporting realist reviews (Wong, Greenhalgh, Westhorp, & Pawson, 2014) and realist evaluations (Wong et al., 2016). These realist approaches view causation as a generative process, where outcomes are caused by context sensitive mechanisms (Pawson et al., 2005). Paramedics working in primary care are conceptualised as a complex intervention that has outcomes which are context sensitive. Therefore, our research approaches will enable us to identify and understand the contexts in which the outcomes of paramedics working in primary care may or may not be effective.

The realist review is able to synthesise a range of relevant data such as quantitative, qualitative and mixed-method research, as well as grey literature. Realist reviews move beyond a description of literature by using a theory-driven, interpretative approach to analysis data from such diverse sources. Findings from our realist review are transferable as we will focus on the mechanisms that cause particular outcomes within the paramedic role in primary care. This will enable us to produce recommendations likely to be useful across the NHS.

A realist evaluation is a form of primary research that is theory-driven. We will collect primary data from ‘real world’ NHS practice to further develop the programme theory that we have developed from the realist review (Pawson & Tilley, 1997). Realist evaluation is important for this project as it recognises the complexity of the real-world working lives of paramedics – who will (by nature of their experience) work differently and in different settings. The approach is ideally suited, as we already know that paramedics are trained to different standards and work in a different capacity in different environments. Therefore, realist evaluation allows us to explore these different possible contexts, how they influence outcomes and through which mechanisms. We will also look at how much patients, healthcare practitioners and the health service in general are benefiting (or not) within these different contexts.

### Patient and public involvement

The realist approach protocol incorporates iterative cycles of engagement with the literature and with the Stakeholder Group. Our stakeholders comprise individuals involved in working in primary care, including paramedics and GPs, patients and organisations including the College of Paramedics and Health Education England. These individuals were identified through professional networks of the authors, or a call for input on social media, and invited to have an informal conversation about their thoughts on paramedics working in primary care. These engagement activities have facilitated the unique provision of advice, feedback and diverse perspectives regarding considerations and outcomes for this evaluation.

As this research develops, we will continue to engage at regular intervals with this stakeholder group to build our understanding of how mechanisms operating at the individual, group, professional and wider workforce level produce context dependent outcomes from paramedics working in primary care.

### Work Package 1: Development of programme theory through a realist review

#### Step 1: Initial programme theory

A separately undertaken systematic scoping review (registered on PROSPERO (ID CRD42018109414)) has already identified theories that begin to explain and develop our understanding of paramedics working in primary care. Some of the findings enabled us to surface underpinning theoretical assumptions regarding certain aspects and processes of paramedics working in primary care (Pawson et al., 2005). These theories were developed by iteratively drawing on the literature from the review, and then consulting with key content experts within the stakeholder group.

This has enabled us to develop an initial programme theory, which has incorporated relevant discussions within the project team and the perspectives, considerations and advice from the stakeholder group.

#### Step 2: Literature search

Step 2 involves formal searches informed by our initial programme theory. The goal is to identify extant literature that will be able to further inform the development of a more detailed programme theory. The process of defining, piloting and conducting formal searches will be done with the support of an information specialist.

The use of the following databases is anticipated: COCHRANE, MEDLINE, CINAHL, PsycINFO, EMBASE, NHSEED, ERIC, DARE, JBI, EBP and OpenGrey. Forward citation searches and backwards chaining will also be undertaken. Terminology and search structure will be informed by Step 1, and any change in the search terms and subject headings will be documented and implemented across databases.

Grey literature, such as policy, stakeholder analysis, reports, conference proceedings, websites, news articles, leaflets and social media that offer useful contextual and/or conceptual information, will be used.

#### Step 3: Data screening

All screening will be undertaken by GE. This will be undertaken in two phases – firstly by title, abstract and keywords. The following inclusion criteria will be used to determine if a document is likely to contain relevant data:

- Population: Our population for analysis will be paramedics. By paramedics, we mean any healthcare professional registered as a paramedic within the United Kingdom, or equivalent clinician in countries within the Organisation for Economic Co-operation and Development (OECD) (e.g. Canada).
- Contribution: We will identify relevant literature studying the contribution of paramedics within primary care settings. We will focus (where available) on factors such as:
  - role;
  - type of intervention being delivered;
  - interaction with other health and social care services;
  - patient and carer satisfaction;
  - clinician satisfaction; and
  - costs.
- Setting: We will seek studies that have been conducted in a primary care setting (e.g. general practitioners, community pharmacists, opticians, dentists). We will include out-of-hours general practice, minor injury units and walk-in centres.

During the screening, documentation relating to clinicians not employed or registered as paramedics will be excluded from this review.

The second phase will review the full texts of the initially screened documents, again alongside the inclusion and exclusion criteria.

A random 10% sample of the retrieved citations will be allocated and reviewed independently by GW to ensure consistency in the screening process. Any ‘disagrees’ regarding citations will be discussed between GE and GW, and for issues that cannot be resolved, they will consult the wider project team for help with coming to a final decision.

Additional searching may be undertaken if there are gaps in the data identified during refinement of the programme theory. If these searches are undertaken, any new or refined inclusion and exclusion criteria will be discussed with the wider project team.

#### Step 4: Selection and extraction

The full-text documents that have ‘passed’ the inclusion and exclusion criteria will be considered for definite inclusion within the realist review. Selection for definite inclusion will be related to our judgements on their ‘relevance’ in their contribution to the development and refinement of the programme theory, and (where needed) judgements about the rigour of the methods used to generate the data (Pawson et al., 2005). Documents that relate to the United Kingdom will be prioritised initially for final inclusion and analysis, so that findings are more likely to be transferable to the NHS. Studies from other countries, where paramedics may work in a similar way as in the NHS but in different OECD healthcare systems, will be drawn on later (where needed) to ensure there are no gaps in the programme theory.

Descriptive document characteristics will be extracted into an Excel spreadsheet. Documents for final inclusions will be uploaded into NVivo for data management and coding. Data extraction will be undertaken by GE, with 10% of extracted data reviewed independently by KRM and VW. Discussions regarding disagreements will be shared within the wider team, and the outcomes recorded.

#### Step 5: Data synthesis

In realist reviews, data synthesis uses data to further develop and refine the initial programme theory. This will follow the process set out by Papoutsi et al. (2018), where we will produce a programme theory that links context to outcomes, using the simple analytic ‘rule of thumb’ of context + mechanism = outcome (CMO). In other words, for this outcome that has been reported in an included document, my interpretation is that it was caused by this mechanism, which was ‘triggered’ by these contexts. This level of detail will enable us to understand what contexts need to be in place for desired outcomes to occur. We can then make inferences about what intervention strategies might be needed regarding the education of paramedics, to ‘create’ the contexts needed for paramedics to practise safely in primary care.

During analysis, we will develop codes through inductive (from data in documents), deductive (informed by the programme theory) and/or retroductive (where inferences are made based on interpretations of the data within documents about mechanisms) reasoning (Manzano, 2016; Pawson & Tilley, 2004; Shearn, Allmark, Piercy, & Hirst, 2017).

During analysis and synthesis we will assess the relevance and rigour of the content within the documents, following the process set out by Abrams et al. (2018). Specifically, we will:

- assess if the document contains data relevant to the development of the programme theory;
- assess if data are sufficiently trustworthy to warrant making changes to the programme theory (where needed); and
- make interpretations and judgements about the data and how they might form CMOs, and how these CMOs relate to each other within the programme theory (where needed drawing either within or across included documents).

Interpretive cross-case comparisons within this synthesis will provide an understanding of the relationship between CMOs across different data sources. Synthesising data across different documents in this way is often necessary to compile CMO configurations, as documents do not always articulate all parts of the configuration. For example, comparing documents which have findings on how the paramedic scope of practice in primary care has different impacts in different settings will enable us to understand how context influences the reported findings. Our analysis will use reasoning processes such as juxtaposing, adjudicating, reconciling, consolidating and situating the evidence (Pawson, 2006).

As the review progresses, we will seek input from the stakeholder group, as this will continually refine and test the programme theory (Wong et al., 2014). The active involvement of those working in primary care will improve how the findings support recommendations, and how changes can be made in practice. The stakeholder group will be our ‘critical friends’, providing us with feedback and advice, as well as sense-checking our findings. To enable this process, we will share with them our emerging findings. If they raise issues that have not been covered in our findings, we will return to the included documents to seek out data to confirm, refute or refine our programme theory (if needed) in response to their feedback.

Review of the programme theory will be undertaken in this way until no new information is provided by the stakeholder group or those paramedics working in primary care, reaching theoretical saturation. The final refined programme theory will be presented in a narrative form, underpinned by a description of the realist synthesis (along with illustrative data that support my realist analyses), and will be summarised using an infographic.

### Work Package 2: Testing of programme theory using empirical data from a realist evaluation

Realist evaluation is a form of primary research that is theory-driven. We will collect primary data from ‘real world’ NHS practice to further develop the programme theory developed from the realist review (Pawson & Tilley, 1997). Taking a realist evaluation approach is important for this project as it recognises the complexity of the real-world working lives of paramedics – who will (by nature of their experience) work differently and in different contexts/areas. The approach is ideally suited, as we already know that paramedics are trained to different standards, and work in a different capacity in different environments. Therefore, realist evaluation allows us to explore these different contexts, how they influence outcomes and through which mechanisms. We will also look at how much patients, healthcare practitioners and the health service in general are benefiting (or not) within these different contexts.

Realist evaluation may be carried out using more than one method to collect relevant data (Ebenso et al., 2017; McEvoy & Richards, 2006; Nurjono et al., 2018); therefore, data collection will occur in two steps: a survey, and focused observations and interviews.

#### Step 1: Survey

Supported by the College of Paramedics, a national cross-sectional survey will focus on gaining information relating to variation in employment conditions, scope of practice, educational requirements and salary for paramedics working in primary care. This approach is a useful way to test specific aspects of the programme theory from the realist review and will provide information on how far this theory reflects the reality of paramedics working in primary care (Kelley, Clark, Brown, & Sitzia, 2003). The precise content of the survey will be informed by the programme theory. Based on current content knowledge of the project team and the initial results of the scoping review, we will likely collect information on gender, region of work, scope of practice, education and salary. The survey will first be piloted and refined using members of the stakeholder group.

##### Sample

Of 25,000 registered paramedics (Health and Care Professions Council, 2018), 11,470 are members of the College of Paramedics (as the professional body) (College of Paramedics, 2019), and it is estimated that 5% of these members work in primary care roles. Based on similar studies (Drennan et al., 2014), an appropriate sample size of 410 is needed to give 80% power at a significance level of 5% for statistical analysis.

The survey will also target formal and informal networks of paramedics working in primary care, in order to reach those who may not be members of the College of Paramedics. This will help to minimise any sampling bias. To increase response rates, up to three reminders will be sent.

##### Data analysis

Given the results of the initial scoping review, it is likely that the data derived will be mixed, and the subsequent quantitative results are more likely to be non-parametric data. If this is correct, then the chi-squared test will be the most appropriate statistical hypothesis test to analyse any group differences within the results.

#### Step 2: Focused observations and interviews

Focused observation of paramedics working in primary care will be undertaken, followed by semi-structured interviews. The contents of the interview will be informed by the programme theory developed from the realist review. Semi-structured interviews will also be conducted with other healthcare professionals in general practice, such as general practitioners, nurses and pharmacists, as well as patients who have received care from a paramedic in primary care. It is important that this evaluation gathers information from the wider healthcare workforce, in order to fully understand the potential impacts of paramedics working in these settings. These interviews will focus around the experiences of working with a paramedic and the care received by the patient.

##### Sample

It is expected that paramedics will work differently in different contexts. Potentially important programme contexts include organisational, geographical, political, economic and social constructs (Greenhalgh et al., 2015). A case study approach will be used to select settings (where paramedics work) informed by the programme theory from the realist review. It is expected that in each of these primary care settings different contexts will affect how and why paramedics practise and the outcomes obtained. Therefore, the sample will include a range of sites to enable collection of relevant data on the variation of practice. Based on similar work investigating roles of healthcare professionals in primary care (Drennan et al., 2014), it is expected that the contribution of 40 individuals across four sites will provide rich enough data to contribute to programme theory development and refinement. Interviews will be split equally across each site, although fewer interviews at each site may be needed if theoretical saturation is reached.

##### Data analysis

Data from interviews are considered as ‘evidence for real phenomena and processes’ (Maxwell, 2012: 103). The programme theory from the realist review will be iteratively ‘retested’ against additional information gained within each interview (Manzano, 2016). Field notes from the focused observations and transcripts from the interviews will be coded within NVivo. The data will be analysed using the same realist logic as used for the realist review (Manzano, 2016).

Consultation will continue with the stakeholder group throughout Work Package 2. For validation, potential CMO configurations will be proposed and discussed with this group, after which the programme theory will be refined to highlight the role of paramedics in primary care, by detailing what their role entails (outcome), how they work (mechanism) and under what conditions (contexts).

## Ethics and dissemination

University of Oxford ethics committee approval will be sought and, as this study will involve NHS staff, Health Research Authority approval will be required before the evaluative aspect of the study begins.

For Work Package 2, written informed consent forms will be obtained from all participants who are engaging with the survey and interview. To maintain confidentiality, no identifiable information from the participants will be obtained and data relating to the participants (such as type of healthcare professional) will be recorded using an encrypted device and later anonymised and transcribed.

Ensuring that the outputs of this project are useful for the development of best practice within the educational development of the paramedic in primary care is a key priority for us. Therefore, a range of outputs will be produced to target a range of audiences:

1. Academic publications in high-impact peer-reviewed journals, as well as professional and academic conferences.
2. Stakeholder consultation and presentation will hopefully see the adoption of the proposed outputs of this research (an education pathway, curriculum and workplace-based tool) into the paramedic postgraduate curriculum (College of Paramedics) or endorsement as a tool for practice (Health Education England).
3. Plain English summaries will provide meaningful summaries of this research as well as ensure engagement with a wide audience range, including patients, paramedics, general practitioners, educationalists and commissioners. These will provide evidence-based sources to inform the practice and implementation of paramedics in primary care.

## Strengths and limitations of this study

- Undertaking a realist review enables us to understand the complexity of how paramedics undertake their role working in primary care, and accounts for the different outcomes they cause under varying contexts – making our work transferable.
- Using a theory-driven realist evaluation approach will ensure findings from this study are able to generate contextually relevant evidence to understand the ways in which paramedics impact on the primary care workforce.
- Stakeholder engagement during programme theory development will ensure a range of perspectives are included, aiding the review’s relevance for other professionals.
- The largest barrier to development, adoption and implementation of the results is the complexity of the topic area. A realist approach is an increasingly acknowledged way of reviewing, evaluating and understanding a complex system, and care and effort will be required to ensure that the outputs are transferable.

## Acknowledgements

The authors would like to thank our Stakeholder Group for their time and contributions to the refinement of our protocol.

## Author contributions

GE, KRM and GW conceptualised the study. GE designed and wrote the protocol manuscript, and all authors contributed to protocol development. GW provided methodological advice. All authors read and approved the final manuscript.

## Conflict of interest

KRM is Chair and GW is Deputy Chair of the United Kingdom’s National Institute of Health Research Health Technology Assessment Primary Care Panel.

## Ethics

Formal ethical approval is not required for the review, as it is secondary research. Formal ethical approval will be sought for WP2 Evaluation.

## Funding

None.
